# Progression of logopenic variant primary progressive aphasia to apraxia and semantic memory deficits

**DOI:** 10.1186/1471-2377-13-158

**Published:** 2013-11-01

**Authors:** Michitaka Funayama, Yoshitaka Nakagawa, Yoko Yamaya, Fumihiro Yoshino, Masaru Mimura, Motoichiro Kato

**Affiliations:** 1 Department of Neuropsychiatry, Ashikaga Red Cross Hospital, Ashikaga-City, Japan; 2 Department of Rehabilitation, Edogawa Hospital, Tokyo, Japan; 3 Department of Neurology, Edogawa Hospital, Tokyo, Japan; 4 Daigo Hospital, Kyoto, Japan; 5 Department of Neuropsychiatry, Keio University School of Medicine, Tokyo, Japan

**Keywords:** Logopenic variant primary progressive aphasia, Apraxia, Semantic memory deficit, Alzheimer’s disease

## Abstract

**Background:**

Due to the nature of neurodegenerative disorders, patients with primary progressive aphasia develop cognitive impairment other than aphasia as the disorder progresses. The progression of logopenic variant primary progressive aphasia (lvPPA), however, has not been well described. In particular, praxic disorders and semantic memory deficits have rarely been reported.

**Case presentations:**

We report three patients in the initial stage of lvPPA who subsequently developed apraxia in the middle stage and developed clinically evident semantic memory deficits in the advanced stages.

**Conclusions:**

The present case series suggests that some patients with lvPPA develop an atypical type of dementia with apraxia and semantic memory deficits, suggesting that these cases should be classified as a type of early-onset Alzheimer’s disease.

## Background

Primary progressive aphasia (PPA) is an initial clinical presentation of degenerative dementia, which is characterized by three variants of progressive language disorder: non-fluent/agrammatic, semantic, and the newly recognized logopenic subtypes [1]. The clinical presentations of the subsequent and advanced stages of non-fluent/agrammatic primary progressive aphasia (naPPA) and semantic variant primary progressive aphasia (svPPA) have been well documented. The later behavioral changes in naPPA are similar to those of frontotemporal dementia and include a decline in social interpersonal conduct, impairment in regulation of personal conduct, emotional blunting, and loss of insight [2]. Patients with svPPA also demonstrate behavioral changes such as loss of sympathy and empathy, narrowed preoccupations, and parsimony [2]. In short, patients with these variants of PPA may develop social behavioral problems in the advanced stages. However, the progression of logopenic variant primary progressive aphasia (lvPPA), the third variant of PPA, has not been well described.

The notable clinical characteristics of patients with lvPPA in the early stage are length-dependent impaired repetition, phonological errors, and anomia. However, the characteristics of patients with lvPPA in the middle and later stages have not been fully studied. As a case series study, Gorno-Tempini et al. 2008 [3] reported that patients with lvPPA showed relative sparing of their functional status 5 years after the onset of symptoms. Caffarra et al. 2013 [4] presented a patient with lvPPA who developed jargon aphasia as a late feature. Rogalski et al. 2011 [5] suggested that the unique linguistic features of lvPPA in the early to middle stage may lose their distinctiveness from other types of PPA as the degeneration worsens. Thus, cognitive impairment other than aphasia has rarely been reported during the deterioration process in lvPPA. However, due to the nature of neurodegenerative disorders, patients with lvPPA inevitably develop cognitive impairment other than aphasia as the disorder progresses.

In the present study, we describe three patients with lvPPA in the early stage who later presented with apraxia in the middle stage and then showed clinically evident semantic memory deficits in the advanced stages. We focused on the clinical and neuropsychological features during cognitive decline in these three patients with lvPPA.

## Case presentations

### Patient 1

**Figure 1:**
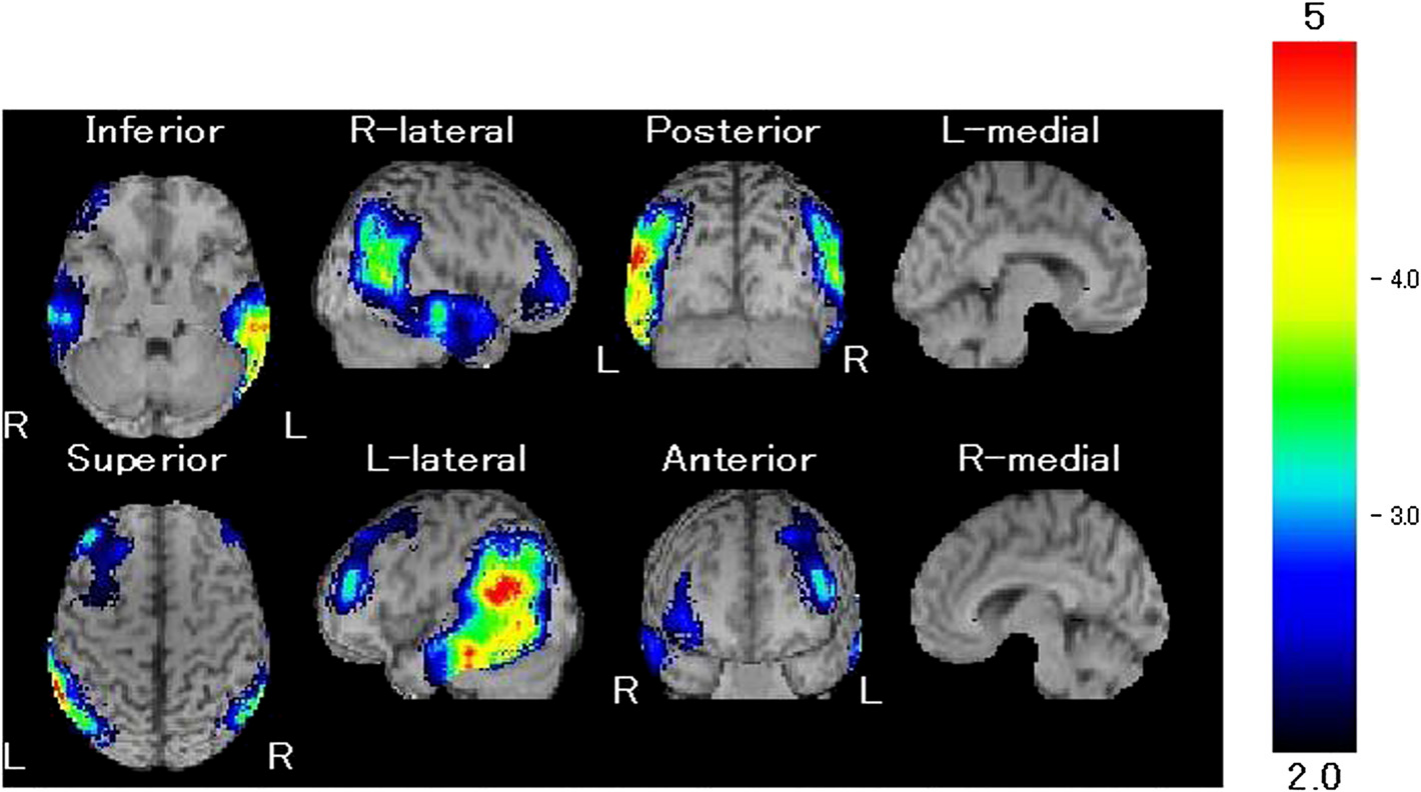
**Brain SPECT eZIS analysis of case 1.** This series of scans showed relative hypoperfusion mainly in the bilateral temporo-parietal areas.

A right-handed woman with 16 years of education began to experience progressive word-finding difficulty at the age of 56 years old. She had been an efficient housewife for more than 30 years. She had shown no previous abnormal neurological or psychiatric abnormalities. She had no family history of dementia. She was referred to our hospital for evaluation of speech difficulties 1 year after onset of the aphasia. Her cranial nerves, motor systems, sensory systems, and coordination systems were normal, and she showed no extrapyramidal signs. Brain magnetic resonance imaging (MRI) and electroencephalography (EEG) were normal. Her laboratory examinations were normal including levels of vitamins, folic acid, and thyroid hormones. The absence of human immunodeficiency virus, syphilis, collagen disease markers, anti-phospholipid antibody, and antineutrophil cytoplasmic antibody-associated vasculitis was noted. Her cerebrospinal fluid was normal. We evaluated the patient with 99mTc-ethylcysteinate dimer single photon emission computed tomography (Tc-99 m ECD SPECT), which was analyzed with an easy Z score imaging system (eZIS) [6] (Figure 1). The image demonstrated hypoperfusion in the bilateral temporo-parietal areas, in particular, the left temporo-parietal junction and left middle and inferior temporal gyri.

Patient 1 showed word-finding difficulty and occasional phonological paraphasia (Table 1), for example, saying 'dansha’ for 'densha’ (train). Comprehension of single words was nearly intact, whereas comprehension of complex sentences was impaired. In addition to spoken language, her comprehension of written language was also impaired for complex sentences. Her writing skills were also impaired. Although grammar and articulation were preserved, her repetition was limited to two words. Her forward digit span was a maximum of three digits. For the backward digit span, she was unable to give any correct answers even for two digits. Non-word repetition deteriorated as the number of morae increased, with 14 correct answers of 14 non-words with two morae, 12 correct of 14 with three morae, 8 correct of 14 with four morae, and 4 correct of 14 with five morae. Her repetition performance including the word-length effect was highly suggestive of a verbal short-term memory deficit. Her ability to calculate was also impaired.

**Table 1:** Demographics and initial linguistic assessment

	**Case 1**	**Case 2**	**Case 3**
Age at onset, years/gender	56/F	53/M	51/M
Education (years)	16	12	12
Years of follow-up	9	7	10
Clinical Dementia Rating total (0–3) for the first 2 years	0.5	0.5	0.5
Forward digit span	3	2	3
Backward digit span	None	None	2
Non-word repetition	Word-length effect	Word-length effect	None
Apraxia of speech	Not found	Not found	Not found
Dysarthria	Not found	Not found	Not found
Phonological paraphasias	Occasional	Frequent	Frequent
Confrontation naming in SLTA, % correct	65	40	35
Word repetition in SLTA, % correct	100	80 (due to paraphasia)	80 (due to paraphasia)
Sentence repetition in SLTA, % correct	20	20	0
Auditory single-word comprehension in SLTA, % correct	100	100	100
Auditory complex sentence comprehension commands in SLTA, % correct	30	0	20

SLTA: Standard Language Test of Aphasia.

However, her orientation and episodic memory were well preserved. Insight, judgment, behavior, concern with hygiene, and effectiveness in performing customary daily activities were also preserved. She continued to live independently. She was partly aware of her aphasia and was frequently frustrated by her predicament. Diagnosis of lvPPA was made according to the clinical features and neuroradiological findings.

Her aphasia gradually worsened, with additional problems starting at age 58 (Table 2). She began to complain of difficulty using electrical appliances such as a landline phone, cell phone, TV remote control, hot water dispenser, and coffee maker. She clearly understood what these appliances are and what they are used for, but she was not sure how to use them. She was unable to drive a car as she did not know how to manipulate the many buttons, the pedals, the steering wheel, and the levers. She was not sure how to heat her bath. Mild ideomotor apraxia of the left hand was found when asked to imitate the examiner’s gesture.

**Table 2:** Clinical course of apraxia and semantic memory impairment following symptom onset and initial radiological assessment

	**Case 1**	**Case 2**	**Case 3**
Ideomotor apraxia	2 years later	2 years later	4 years later
Conceptual apraxia	4 years later	5 years later	8 years later
Episodic memory deficit	4 years later	3 years later	7 years later
Inability to recognize family members or relatives	7 years later	5 years later	9 years later
Pica	7 years later	6 years later	9 years later
Relative hypoperfusion on SPECT	Bilateral temporo-parietal area	Bilateral temporo-parietal area	Bilateral temporo-parietal area

SPECT: single photon emission computed tomography.

At age 59, her inability to use electrical appliances extended to tools for everyday life. She was no longer able to use a nail clipper or open a suitcase. At age 60, she presented with remarkable semantic errors when using tools. When she tried to boil water, she used a pan instead of a kettle. She tried to unlock her car door with a remote control for the air conditioner. She brushed her teeth with a comb.

With the Standard Language Test of Aphasia (SLTA) in Japanese [7], her correct naming decreased from 65% at age 58 to 10% at age 60. Although her comprehension of single words remained 100% even at age 60, her comprehension of command sentences fell from 30% at age 58 to 0% at age 60. To study her ability to use tools, she was given 20 tools for use in everyday life, such as scissors and comb, and asked to show how they are used. She used all 20 objects correctly at age 59. However, her performance worsened to 17 correct of 20 at age 60, 10 correct of 20 at age 61, and 5 correct of 20 at age 62. Errors were found mainly in how to manipulate and even how to hold an object in the later stage.

It was not until age 60 that she showed episodic memory deficits and behavioral changes such as excitement and aggression. At age 61, her ability to copy the interlocking pentagons of the Mini-Mental State Examination [8] was poor, and the number of correct answers in the Benton Visual Retention Test [9] was 2 out of 10 designs, suggesting that she had visuospatial deficits and/or visual memory deficits. She could not perform the motor series Luria test of the frontal assessment battery [10] with her right hand, suggesting her frontal function had also deteriorated. At age 63, she was unable to recognize her husband. She began to frequently eat non-edible objects such as diapers.

### Patient 2

A right-handed man with 12 years of education experienced a gradually progressive speech disorder and difficulty in using a personal computer at age 53. Although he had worked for a manufacturing company for 35 years after high school, he became unemployable in his capacity as a factory worker. Subsequently he found a job as a janitor, but he was fired within a year because of his word-finding difficulty. At age 54, he was referred to our hospital for evaluation of his problems. He had no previous medical or psychiatric history. Regarding his family history, one of his older brothers had been diagnosed with probable Alzheimer’s disease. His physical and neurological examination revealed no remarkable findings. His blood test and serum chemistry, which covered many of the same items as in Patient 1, were normal. His EEG was normal, but brain MRI showed enlargement of the bilateral sylvian fissures. N-isopropyl-p[^123^I] iodoamphetamine (IMP) SPECT with three-dimensional stereotactic surface projections (3D-SSP) analysis [11] showed relative hypoperfusion in the bilateral temporo-parietal areas, extending from the inferior parietal lobule to the temporo-parietal junction to the middle and inferior temporal gyri.

His main difficulties were related to word finding and frequent phonological paraphasias (Table 1). Furthermore, he frequently presented with phonological retrieval difficulty, as he spoke fragmentary syllables as in 'tochi’ for 'tomodachi (friend)’, and 'sen’ for 'sensha’ (military vehicle). No anarthria or dysarthria were noted. His grammar was preserved. His forward digit span was limited to two digits, and he was unable to perform the backward digit span. In non-word repetition, a word-length effect was noticed, with 11 correct answers of 14 with two morae, 6 correct of 14 with three morae, 3 correct of 14 with four morae, and 1 correct of 14 with five morae. These clinical findings suggested a verbal short-term memory deficit. Comprehension of single words remained intact with 100% correct in the SLTA, whereas comprehension of complex sentences was severely impaired. His reading abilities and his writing skills were also poor. However, orientation and episodic memory were intact at this time. No behavioral abnormalities were noted. He continued to live alone independently. He was partly aware of his aphasia and was easily irritated by his word-finding difficulty. Diagnosis of lvPPA was made according to the clinical and neuroradiological findings.

At age 55, ideomotor apraxia of both hands was observed when asked to imitate the examiner’s gesture (Table 2). The phenomenon of body parts as objects was frequently noted. Apathy became gradually evident, and his family sometimes had to supervise his daily living.

At age 56, he became forgetful and sometimes became lost. He was also unable to open and shut off the gas valve. He had particular difficulty in using electrical appliances in daily life. At age 57, his speech rate had significantly slowed down due to difficulty with lexical and phonological retrieval. His comprehension of single words gradually worsened, and he scored 80% correct on the SLTA. His reading and writing skills also deteriorated to the point that he was unable to write his own name. His ability to copy the interlocking pentagons of the Mini-Mental State Examination was poor, and the number of correct answers in the Benton Visual Retention Test was only 1 out of 10 designs, suggesting that he had visuospatial deficits and/or visual memory impairment.

At age 58, he presented with “TV sign” [12], in which he thought that people on TV were in his room, and he tried to speak to them. He was unable to use almost all the tools of everyday life. He tried to use a toothbrush to comb his hair. He would occasionally shout inexplicably. He was no longer able to recognize his relatives. At age 59, he started to eat waste thread and rubbish with crumbs on the floor.

### Patient 3

A right-handed man with 12 years of education noticed progressive speech difficulties at age 51. At age 52, he was no longer able to read the clock and found himself sometimes unable to comprehend long sentences. Although he had been employed as a salesman in an apparel business for more than 30 years, he was fired due to his aphasia. He had no previous medical or psychiatric problems. He had no family history of dementia. His physical and neurological examinations were normal. The patient underwent numerous laboratory tests, which were normal, including blood tests and serum chemistry. His EEG was normal. Brain MRI showed enlargement of the bilateral sylvian fissures. Brain IMP SPECT 3D-SSP analysis showed relative hypoperfusion in the bilateral temporo-parietal areas.

One year after symptom onset, examination showed progressive aphasia with word-finding difficulties, frequent phonological paraphasia, and mild word deafness (Table 1). Speech repetition was limited to a single word. Non-word repetition was almost impossible to execute due to phonological paraphasia. His phonological paraphasia for repetition or word retrieval involved omission, substitution, rearrangement, and addition of speech sounds. His forward digit span was limited to three digits, and his backward digit span was a maximum of two digits. His articulation and grammar were preserved. Comprehension of both spoken and written words was intact. Comprehension of complex sentences, however, was impaired in both auditory and visual conditions. His writing skills were also impaired. He was somewhat aware of his aphasia. Although he was often irritated by his aphasia, he continued to live a nearly independent life. His clinical features and neuroradiological findings led to a diagnosis of lvPPA.

Over the years, his aphasia slowly but definitely worsened. With SLTA, correct naming decreased from 35% at age 52 to 0% at age 55. Comprehension of single words also fell from 100% at age 52 to 70% at age 55. The characteristics of his paraphasia at this point were occasional neologistic jargon and remarkable phonological addition errors. For example, he said 'takenokokonoko’ for 'takenoko (bamboo)’, a phenomenon that can also be regarded as logoclonia. Cognitive deficits in addition to aphasia became evident at age 54 and progressively deteriorated. At age 55, he began to have problems in daily life. He forgot how to use electrical appliances. He developed ideomotor apraxia of both hands when instructed to demonstrate how to use tools or to imitate the examiner’s actions. He became partly dependent on his family.

At age 58, most of his speech consisted of logoclonia such as “sonnnanananananana” and “sirorourorororo” and he was no longer able to comprehend most single words. He was deeply frustrated by his aphasia and sometimes became aggressive. At age 59, he started to eat food with his fingers because he was no longer able to use chopsticks or a spoon. Other tools he was unable to use included a shaver and a toothbrush. He seemed to understand what these tools were used for when his choice for each action was accompanied with the proper tools. However, at age 59, he gradually became confused with what to use for a desired action. He confused toothpaste with shaving cream. His ability to copy the interlocking pentagons of the Mini-Mental State Examination was poor, and the number of correct answers in the Benton Visual Retention Test was only 2 out of 10 designs, suggesting that he had visuospatial deficits and/or visual memory impairment.

At age 60, he started to glare at everyone, as he was unable to recognize family members. He would try to use and manipulate whatever objects he saw. However, he never understood what these objects were and sometimes put them in his mouth and tried to chew them. In addition, he ate cat food several times.

### Case summary

Although all three cases exhibited some cognitive impairment other than aphasia at initial presentation such as acalculia, inability to use a computer, and inability to read a clock, their main symptom was limited to the language impairment of lvPPA, which began in their 50s. One of the three cases had a family history of probable Alzheimer’s disease. Their Clinical Dementia Rating scale [13] remained at 0.5 for the initial 2 years of their illness. Their aphasia progressively worsened, and each patient showed progressive cognitive impairment, which became evident in 2 or more years after the onset of symptoms. The characteristics of their subsequent cognitive impairment were apraxia, which extended from ideomotor (praxis production deficits) to conceptual apraxia (impaired knowledge for tool-action or tool-object associations as well as mechanical knowledge). These apraxias presumably accounted for the patients’ difficulties in using electrical appliances. Episodic memory deficits as well as visuospatial deficits became apparent several years after the onset of symptoms. Lastly, clinically evident semantic memory deficits were noted, including a lack of recognition of their family members or relatives. Their primary affected areas involved a large portion of the bilateral temporo-parietal lobes, extending from the inferior parietal lobule to the temporo-parietal junction to the middle and inferior temporal gyri.

## Conclusions

The present case series provides new insight into the progression of lvPPA. The three right-handed patients with lvPPA developed language decline with subsequent apraxia, followed by clinically evident semantic memory deficits. The results suggested that some patients with lvPPA develop an atypical type of dementia with apraxia and semantic memory deficits.

The clinical course of lvPPA in this study differs from the other variants of PPA, i.e., naPPA and svPPA, which result in profound social and behavioral problems. In contrast, in the present cases, the activities of their social lives were well preserved until the later stage. The patients also had some awareness of their illness. Although semantic memory deficits were noted, similar to the deficits in svPPA, the clinical courses of the two variants were quite different from each other. In particular, apraxia was a prominent feature in the present lvPPA cases, including ideomotor and conceptual apraxia along with difficulties in using home electrical appliances.

The outcomes of the present cases differed from those of previous lvPPA reports. Although a previous report described almost pure aphasia [3], the present cases developed further cognitive decline. Several possibilities could be postulated to account for the different clinical courses. First, the duration of the follow-up period of the present cases was longer than for the previous report. Gorno-Tempini et al. [3] acknowledged that extensive follow-up was not available in most patients with lvPPA that they examined. Given the nature of degenerative disorders, a variety of types of cognitive impairment in addition to aphasia inevitably develops in the advanced stages. Second, the affected brain regions may have been different. The main lesions in our cases extended to the bilateral temporo-parietal areas, whereas the previous cases showed only a unilateral left lesion. In addition, the affected area in our cases involved a large portion of the temporo-parietal areas, and may have involved a larger area than that of previous reports.

Apraxia has been reported in degenerative diseases. Although apraxia in corticobasal ganglionic degeneration is well known, other types of degenerative diseases are also regarded as involving apraxia [14,15], in particular, Alzheimer’s disease. Ideomotor apraxia [16-20] and conceptual apraxia [21-24] have frequently been noted in Alzheimer’s disease. In contrast, in svPPA or Pick’s disease, apraxia has been documented in only two case reports [25,26]. Bilateral hypoperfusion on SPECT is not suggestive of corticobasal ganglionic degeneration, which is characterized by striking asymmetric atrophy and/or hypoperfusion. Moreover, the present cases had no signs of parkinsonism, which rules out the possibility of corticobasal ganglionic degeneration. Instead, the onset of Alzheimer’s disease may reasonably account for the apraxia observed in our cases.

Although semantic memory deficits are a hallmark of svPPA, these deficits have been described in Alzheimer’s disease as well [27-31]. Some researchers have pointed out that semantic memory deficits in Alzheimer’s disease can be regarded as a continuum beginning with conceptual apraxia [23,24]. According to Dumont et al. 2000 [23], the representations of action-semantic and semantic knowledge of objects are often simultaneously disrupted in Alzheimer’s disease, suggesting that conceptual apraxia and semantic memory deficits are closely related. Falchook et al. 2012 [24] shared a similar view, suggesting that semantic taxonomic relationships correlate with performance on tests for conceptual apraxia in people with Alzheimer’s disease. The coexistence of conceptual apraxia and semantic memory deficits in our cases is highly suggestive of the onset of Alzheimer’s disease.

The areas of the brain that are primarily affected in the present cases may account for the development of apraxia and semantic memory deficits. The main location of lesions in lvPPA, but not naPPA or svPPA, involves parietal areas, which have been associated with various types of apraxia [32]. Primary progressive ideomotor apraxia in degenerative diseases is also considered to involve posterior parietal lesions [33]. From an anatomical point of view, lvPPA may develop into or be accompanied by apraxia during the course of the illness. The extension of the affected area to the middle and inferior temporal gyri, which was found in our cases, is thought to be related to semantic memory deficits. Recent neuroimaging studies have demonstrated that not only the anterior temporal area [34], but also the middle and inferior temporal gyri, are crucial for semantic memory [35,36]. The different neural substrates for semantic memory may reflect category-specific semantic systems. Although previous cases with deficits for living things often resulted from anterior temporal lobe damage [37], deficits for non-living things or man-made objects have been reported after left temporo-parietal damage [38]. From a lesion study involving lexical retrieval, words for living things are associated with the left anterior temporal lobe, whereas those for tools are associated with the left posterior temporal lobe [39]. Neuroimaging studies have also demonstrated that the left posterior middle temporal gyrus is particularly important for perceiving and knowing about tools and their functions [40]. Because apraxia is substantially related to tools, the conceptual apraxia observed in our cases may have resulted from posterior temporal lobe damage. In this context, semantic memory deficits in patients with lvPPA may involve tools more than living things.

The clinical pictures of the present cases most likely suggest Alzheimer’s disease. The primary locations of the lesions are also consistent with lesions in Alzheimer’s disease, which involve the lateral temporo-parietal areas [41-43] as well as the medial temporal and medial parietal cortex. Furthermore, recent neuropathological studies have shown that most patients with lvPPA have Alzheimer’s disease [3,44,45]. As the conditions of the patients reported here deteriorated relatively rapidly to the point that severe semantic memory deficits were noticed in their late 50s or early 60s, a type of early-onset Alzheimer’s disease [46,47] is suggested, which often features aphasia or apraxia rather than episodic memory deficits as an initial symptom, followed by relatively rapid deterioration.

Some limitations must be considered when interpreting the present cases. First, we did not perform brain autopsies to confirm the clinical diagnoses. Second, the observation that patients with lvPPA developed apraxia and semantic memory deficits may not be surprising, because patients with localized degeneration of parietal regions can show apraxia, and patients with Alzheimer’s disease often develop semantic memory deficits in the advanced stages. However, the unique, previously undocumented progression from lvPPA to apraxia and semantic deficits may be a typical course of deterioration in patients with lvPPA and is quite different from those with svPPA or naPPA. This information may be important in clinical practice.

In conclusion, the present case series suggested that some patients with lvPPA develop an atypical type of dementia with apraxia and semantic memory deficits, indicating that these cases could be classified as a type of early-onset Alzheimer’s disease.

### Consent

Written informed consent was obtained from the patients and their family members for publication of this case series and any accompanying images. A copy of the written consent is available for review by the Editor of this journal.

## Abbreviations

lvPPA: Logopenic variant primary progressive aphasia; naPPA: Non-fluent/agrammatic variant primary progressive aphasia; svPPA: Semantic variant primary progressive aphasia; PPA: Primary progressive aphasia; MRI: Magnetic resonance imaging; EEG: Electroencephalography; Tc-99 m ECD SPECT: 99mTc-ethylcysteinate dimer single photon emission computed tomography; eZIS: Easy Z score imaging system; SLTA: Standard Language Test of Aphasia; IMP SPECT: N-isopropyl-p[^123^I] iodoamphetamine single photon emission computed tomography; 3D-SSP: Three-dimensional stereotactic surface projections.

## Competing interests

The authors declare that they have no competing interests.

## Authors’ contributions

MF acquired case data, designed the study, and drafted the manuscript. YN, YY, and FY acquired case data. MM and MK supervised the study and helped to draft the manuscript. All authors read and approved the final manuscript.

## Pre-publication history

The pre-publication history for this paper can be accessed here:

http://www.biomedcentral.com/1471-2377/13/158/prepub
